# Effects of Vitamin D on Cardiovascular Risk and Oxidative Stress

**DOI:** 10.3390/nu15030769

**Published:** 2023-02-02

**Authors:** Guilherme Renke, Bernardo Starling-Soares, Thomaz Baesso, Rayssa Petronio, Danilo Aguiar, Raphaela Paes

**Affiliations:** 1 National Institute of Cardiology, Brazilian Ministry of Health, Rio de Janeiro 21040-900, Brazil; 2 Nutrindo Ideais Performance and Nutrition Research Center, Rio de Janeiro 22640-100, Brazil; thomazbaesso@gmail.com (T.B.); rayssalucenapetronio@gmail.com (R.P.); daniloaguiar45@hotmail.com (D.A.); raphaelafmp@gmail.com (R.P.); 3 Extreme Sports Nutrition Institute—INEE, Belo Horizonte 31270-901, Brazil; bernardostarling@gmail.com

**Keywords:** cardiovascular, vitamin D, 25-hydroxyvitamin D [25(OH)D], 1,25-dihydroxycholecalciferol [1,25(OH)2D], calcitriol, oxidative stress

## Abstract

Introduction: Vitamin D has been primarily studied as an important factor influencing bone and calcium metabolism. Metabolites of vitamin D are essential for whole-body calcium homeostasis, maintaining serum calcium levels within a narrow range by regulating this process in the bones and gut. Nevertheless, its deficiency is also related to increased risk of type 2 diabetes mellitus (T2DM), metabolic syndrome (MS), and cardiovascular disease (CVD)—with increased visceral adipose tissue and body mass index (BMI), as well as the frequently associated hypercholesterolemia. It has been reported that vitamin D levels are inversely related to cardiovascular (CV) risk in men and women. However, the effects of vitamin D on distinct outcomes in women and the dose of supplementation needed to improve clinical endpoints have not been established. 25-Hydroxyvitamin D [25(OH)D] reduces systemic inflammatory mediators in CVD and favors the release of anti-inflammatory cytokines from the immune system. In addition, 25(OH)D can be primarily converted into calcitriol (1,25-dihydroxycholecalciferol [1,25(OH)2D]) in the kidneys through the action of the 1-α-hydroxylase enzyme. Calcitriol, through the downregulation mechanism of renin expression, renin–angiotensin–aldosterone system (RAAS) activity, and its interaction with the vitamin D receptor, can bring CV benefits. The calcitriol form also lowers parathyroid hormone (PTH) levels by indirectly causing a reduction in aldosterone and mineralocorticoid synthesis. Elevated plasma aldosterone is related to endothelial dysfunction and CVD in hypovitaminosis D status. Conclusion: Vitamin D supplementation may benefit certain risk groups, as it improves metabolic variables, reducing oxidative stress and CV outcomes. More studies are needed to define interventions with vitamin D in men and women.

## 1. Introduction

Recent studies have shown that vitamin D deficiency is a global health problem [1,2,3]. The minimum laboratory value of 25-hydroxyvitamin D [25(OH)D], 20 ng/mL, is not reached by 65% of the world’s elderly population, and deficiency is present even in younger individuals [4]. Vitamin D is one of the main factors influencing bone and calcium metabolism. Its deficiency is also related to a higher risk of type 1 diabetes, type 2 diabetes mellitus (T2DM), metabolic syndrome (MS), coronavirus disease 2019 (COVID-19), and cardiovascular disease (CVD), including arterial hypertension (AH) [1,5,6,7,8,9].

This review article assesses the potential relevance of vitamin D concentrations and their relationships with oxidative stress and cardiovascular (CV) risk. Some evidence suggests that vitamin D levels are inversely related to blood pressure and the risk of AH, based on studies in Western populations [9,10,11]. Animal studies provide strong support for the function of 1,25-dihydroxycholecalciferol [1,25(OH)2D] through the downregulation of renin expression and renin–angiotensin–aldosterone system (RAAS) activity through its interaction with the vitamin D receptor [12]. Vitamin D’s function as a potent negative endocrine regulator of renin gene expression provides insights into the development of AH and how vitamin D deficiency could favor its development or progression [13]. The 25(OH)D is converted to 1,25(OH)2D in the kidneys through the action of the 1-α-hydroxylase enzyme. However, macrophages, endothelial cells, and smooth muscle cells are also capable of performing this conversion. This increase in the local production of active vitamin D acts as an autocrine and paracrine factor, which is critical for specific cell functions [14].

A growing body of evidence from animal and human studies illustrates that vitamin D decreases systemic inflammatory mediators in CVD and favors the release of anti-inflammatory cytokines from the immune system [15,16]. In diabetics, for example, 1,25(OH)2D has been shown to suppress the uptake of cholesterol into the endothelium by macrophages and reverse the increased cholesterol deposition through the downregulation of endoplasmic reticulum activity. Furthermore, this suppression of the endoplasmic reticulum activity shifts M2 macrophages to the M1 phenotype, suggesting that vitamin D’s regulation of endoplasmic reticulum activity may be a potential therapy for patients with atherosclerosis. Macrophages recruited to the subendothelial space respond to environmental signals that dictate their differentiation into different phenotypes. In atherosclerotic plaques, M1 macrophages express membrane receptors that facilitate the egress of immune cells from the atherosclerotic plaque. In contrast, M2 macrophages, induced by interleukins (e.g., IL-4, IL-10), are known to attenuate excessive inflammation, facilitating collagen production and fibrosis [17,18].

In preclinical studies, for example, the absence of vitamin D receptor signaling increased blood pressure by increasing renin release and accelerating atherogenesis, possibly through the local activation of macrophages and the RAAS [18].

## 2. Material and Methods

The presented scientific literature was reviewed and the studies were downloaded from the PubMed (https://pubmed.ncbi.nlm.nih.gov/ (accessed on 1 December 2022)), ScienceDirect (https://sciencedirect.com/ (accessed on 1 December 2022)), and SciELO (https://scielo.com.br/ (accessed on 1 December 2022)) databases. Combinations of several search terms—such as “vitamin D”, “cholecalciferol”, “calcitriol”, “1,25 dihydroxycholecalciferol”, “25 hydroxycholecalciferol”, “CVD”, “cardiovascular”, “antioxidant”, “anti-inflammatory”, “oxidative stress”, “redox”, “hypertension”, “hypercholesterolemic”, and “antihypertensive”—were applied. After the search, the studies were classified according to the health-specific parameters of the text.

## 3. Results

### 3.1. Vitamin D and Its Physiology

Vitamin D is a generic name used to designate a group of fat-soluble compounds of endogenous or exogenous origin that have the physiological characteristics of a hormone [16]. The endogenous form is produced in the epidermis, by the action of ultraviolet B rays, from the activation of 7-dehydrocholesterol (provitamin D3) with the formation of vitamin D3, which undergoes hydroxylation primarily in the liver and in the proximal convoluted tubules of the kidneys, resulting in 1,25(OH)2D or calcitriol, which is the most active form of vitamin D [16].

In humans, exposure to the Sun is responsible for about 90–95% of the supply of vitamin D. The exogenous sources—i.e., food or ergocalciferol—are unimportant, as significant amounts of vitamin D are found only in oily fish and mushrooms cultivated in the Sun. The effectiveness of endogenous production of vitamin D depends on environmental factors related to latitude, season, and the time of day when Sun exposure occurs. Genetic factors, body composition, physical activity, topical use of sunscreens, and skin pigmentation are also important [19,20].

According to consensus, obesity is strongly related to serum levels of 25(OH)D, with a negative correlation between vitamin D levels and body mass index (BMI) [21] that could be explained both by the shorter time of exposure to the Sun—linked to the more sedentary habits of the obese population—and by the reduction in vitamin D’s bioavailability, related to the increase in the storage of vitamin D in the adipose tissue [22]. It has also been proposed that hypovitaminosis D could promote a physiological elevation of parathyroid hormone (PTH) levels by increasing the intracellular calcium concentration in adipocytes which, in turn, would stimulate lipogenesis and weight gain [23].

To clarify this issue, Vimaleswaran and colleagues recently performed a bidirectional Mendelian randomization study that analyzed 21 cohorts with a total of 42,024 patients, demonstrating that genetically determined 25(OH)D levels were not significantly correlated with BMI [24]. The authors concluded that obesity should be seen as an important causal risk factor that accounts for approximately one-third of cases of vitamin D deficiency. Thus, the obesity “epidemic” may be contributing to the high prevalence of insufficiency (25(OH)D < 30 ng/mL) and deficiency (25(OH)D < 20 ng/mL) of vitamin D according to criteria established by the Endocrine Society, currently observed in almost half of the world’s population [25]. The recommendation for vitamin D supplementation includes the following criteria: 400–1000 IU/day, 600–1000 IU/day, and 1500–2000 IU/day for infants up to 1 year of age, children up to 18 years of age, and adults, respectively. The Endocrine Society also states that obese adults require 2–3 times more vitamin D than eutrophic adults [26].

From a physiological point of view, the active form of vitamin D increases intestinal calcium absorption and extracellular calcium concentration, indirectly suppressing PTH secretion, in addition to having a direct inhibitory effect on gene transcription in the parathyroid glands. In vitamin D deficiency, there is an elevation of PTH and, consequently, an increase in renal tubular reabsorption of calcium and its mobilization from the bone through osteoclastic stimulation [27].

The main functions of vitamin D are related to calcium/phosphorus homeostasis and the promotion of bone mineralization. However, considering that the vitamin D receptor coding gene transcription can regulate about 3% of the human genome, and that vitamin D receptors are present in several tissues (e.g., parathyroid cells, renal tubules, pancreatic β cells, immune system cells, neural cells, cardiac musculature, the muscular layer of vessels and the endothelium), many studies have suggested that vitamin D deficiency may contribute to the pathogenesis of conditions such as autoimmune diseases, cancer, COVID-19, glucose and lipid alterations, AH, MS, endothelial dysfunction, and CVD [2,7,8,9,28,29,30,31,32,33]

Evidence demonstrates that a low concentration of 25(OH)D is considered to be a risk factor for chronic diseases and is also related to all-cause mortality [34]. Thus, it is expected to increase morbidity and mortality for diseases associated with vitamin D deficiency in the coming years [35]. Evidence on the non-skeletal health benefits of vitamin D has gained momentum in recent years, although guidelines still only support its benefits for skeletal outcomes [34].

Vitamin D deficiency has been associated with several conditions and diseases, including immunological pathologies, cancer, and COVID-19. In fact, recent research has supported the hypothesis that vitamin D deficiency is related to the incidence, severity, and mortality attributed to COVID-19 [7,8]. It has been demonstrated that, in men, the administration of 25(OH)D, without neglecting the hypercalcemic risk, can be considered a suitable indication for treating patients with COVID-19 [7]. Furthermore, studies have concluded that individuals with a higher risk of severe COVID-19 correspond to those with a higher risk of severe vitamin D deficiency, including the elderly and obese [8]. However, although a role for vitamin D has been proposed in counteracting COVID-19 through potential modulation of immune dysfunction and cytokine storm, to date, there is still a lack of evidence in the literature in terms of differences in serum levels in large cohorts, according to disease state [9].

In addition, one of the big questions about vitamin D supplementation is whether there are a variety of recommended vitamin D thresholds that are used to study their health effects. An insufficient level of vitamin D—considered to be <50 nmol/L—is most often used to describe hypovitaminosis D in recent studies. Thus, it seems to be difficult to interpret some studies that consider normal values of vitamin D to be between 25 and 49 nmol/L [36].

In chronic diseases such as T2DM, vitamin D supplementation has shown a 10% risk reduction in overweight individuals with pre-T2DM for progression to T2DM. However, this effect was smaller than that produced by the use of oral hypoglycemic agents or lifestyle modifications [37,38]. Additionally, with regard to cancer, there are strong preclinical data linking vitamin D to cell-cycle control. Furthermore, many observational studies have associated low vitamin D status with an increased risk of cancer or poor prognosis. There appears to be a benefit of vitamin D supplementation on cancer mortality, especially when the follow-up is longer than 4 years [37,38,39].

The relationship between vitamin D and stroke has been investigated in some studies, and it has been proposed that vitamin D could be a prognostic biomarker for functional outcomes in stroke patients. However, the evidence is still inadequate to support the effectiveness of vitamin D supplementation in stroke and post-stroke recovery [40].

Vitamin D appears to have an important immunomodulatory effect due to its influence on all cells of the immune system through the production of cytokines. Patients with vitamin D deficiency, after receiving vitamin D supplementation, have a reduced risk of upper respiratory infections. Another documented benefit was in patients with asthma or respiratory disease, where vitamin D supplementation reduced the risk of acute upper respiratory infections and improved expiratory lung function. With regard to autoimmune diseases, there may be a worsening in cases of vitamin D deficiency. This is due to the adaptive immune system being negatively regulated by calcitriol and, therefore, vitamin D deficiency may predispose individuals to autoimmune diseases [41].

Regarding bone metabolism, vitamin D status is related to inadequate bone health—especially in high-risk groups, such as cancer patients. A very low prevalence of patients with adequate bone health and normal vitamin D status was observed in a cohort of women with breast cancer. Thus, bone health and vitamin D status must be appropriately assessed and managed to reduce the risk of fragility fractures in patients with cancer and other chronic diseases [39].

### 3.2. Vitamin D, Oxidative Stress, and Inflammation

Oxidative stress has confirmed its importance in cellular damage through the action of reactive oxygen species (ROS). However, there is a balance between oxidant and antioxidant activity under normal conditions. The antioxidant defense system includes glutathione peroxidase, superoxide dismutase, and various vitamins, including vitamin D, minerals, and other compounds. Vitamin D, in particular, is known to have the capacity to reduce oxidative stress through the positive regulation of cellular glutathione and superoxide dismutase. Several meta-analyses and clinical studies with different groups have demonstrated the important role of vitamin D in the management of oxidative stress (Table 1).

Sepidarkish and colleagues presented a meta-analysis of 13 clinical trials, finding that vitamin D supplementation increased levels of serum total antioxidant capacity (TAC) (SMD: 0.54 mmol/L, 95%CI: 0.29 to 0.79; I2 = 65.4%, *p* = 0.001) and total serum glutathione (GSH) (SMD: 0.33; 95% CI: 0.11 to 0.54, *p* = 0.003; I2 = 61.2%, *p* = 0.001). Malondialdehyde (MDA) decreased significantly (SMD: −0.40 mmol/L, 95% CI: −0.60 to −0.21, *p* < 0.001), while nitric oxide (NO) was not significantly different (0.17, 95% CI: −0.10 to 0.45, I2 = 71.1%, *p* = 0.21). [46].

A comprehensive meta-analysis listed 23 studies in different groups of individuals with different clinical health conditions, genders, and ages, finding improvements in MDA levels (ES = −0.37; 95% CI: −0.48, −0.25, *p* < 0.001) and in the inflammatory markers high-sensitivity *C*-reactive protein (hs-CRP) (ES = −0.42; 95% CI: −0.55, −0.29, *p* < 0.001) and tumor necrosis factor (TNF)-α (ES = −0.27; 95% CI: −0.42, −0.12; *p* < 0.001) at the time of vitamin D supplementation [48].

In specific groups, such as women with polycystic ovary syndrome (PCOS), it was found that vitamin D supplementation led to an increase in TAC, leading to reductions in the MDA levels (−1.64, 95% CI −2.26 to −1.02, *p* < 0.001) and in the oxidative stress and inflammatory marker hs-CRP (−1.03; 95% CI, −1.58, −0.49; *p* < 0.001); however, there were no changes in NO or GSH [44].

In a systematic review including 33 randomized clinical trials of diabetic patients to analyze the relationship of vitamin D supplementation with some markers, there were decreases in hs-CRP (WMD 0.27; 95% CI, −0.35, −0.20; *p* < 0.001) and MDA (WMD − 043, 95% CI −0.62, −0.25, *p* < 0.001), as with the group of women with PCOS. Along the way, there were still significant increases in the release markers of NO (WMD 4.33, 95% CI 0.96, 7.70), GSH (WMD 82.59, 95% CI 44.37, 120.81, *p* < 0.001), and TAC (WMD 57.34, 95% CI 33.48, 81.20, *p* < 0.001) [45].

A randomized controlled study corroborating these findings, also including T2DM patients, performed some interventions including the supplementation of at least 14,000 IU per week (deficient group: initial supplementation was 50,000 IU per week of vitamin D for three months, and then supplementation was reduced to 14,000 IU per week for another three months), finding a decrease in the concentration of ROS at a follow-up period of 6 months and a decrease in the inflammatory marker CRP, although this was not statistically significant [47].

In another study analyzing younger age groups, the authors found similar results. In a Chinese study involving more than 1400 children aged 7 to 11 years, comparing deficiency and sufficiency groups, there was an inverse association of 25(OH)D levels with hs-CRP (1.21 ± 0.13 vs.1.04 ± 0.14), interleukin-6 (IL-6) (3.83 ± 0.33 vs. 3.58 ± 0.34), MDA (2.87 ± 0.21 vs. 2.83 ± 0.27), and superoxide dismutase (SOD) (95.38 ± 12.22 vs. 127.62 ± 15.98 U/mL, *p* < 0.001) [43]. Corroborating these findings, an observational study carried out with 66 obese children found a correlation between vitamin D deficiency and increased levels of oxidative stress and inflammation markers. After partial correlation analysis, it was found that there was an inverse relationship of vitamin D levels with 3-nitrotyrosine (r = −0.424, *p* = 0.001), soluble vascular cell adhesion molecule-1 (sVCAM-1) (r = −0.272, *p* = 0.032), and the inflammation marker IL-6 (18.0 [12.2–25.7] vs. 13.7 [10.7–19.3] pg/mL, *p* < 0.036) [42].

As seen in the studies cited above, chronic low-grade inflammation that is typically characterized by increased levels of *C*-reactive protein (CRP), interleukin-6 (IL-6), and tumor necrosis factor-α (TNF-α) is associated with increased risk of type 2 diabetes mellitus (T2DM) and cardiovascular diseases (CVD). A series of in vitro and in vivo studies have demonstrated that vitamin D supplementation decreases the levels of inflammatory cytokines and increases the concentrations of anti-inflammatory markers [17,42,43,44,45,46,47,48]. Vitamin D and its metabolite can inhibit nuclear transcription factor κB activity by increasing IκB expression and reducing IκB-α phosphorylation. Therefore, vitamin D has the potential to inhibit the production of pro-inflammatory factors (42,44). Thus, vitamin D can decrease the generation of free radicals and inflammatory cytokines, mainly by deactivating NF-κB-dependent pathways, showing its favorable effects on oxidative stress reduction [46].

In summary, vitamin D should be considered as an adjuvant therapy for treating inflammation and increased oxidative stress diseases. More research is still required to determine the dosages and duration of treatment.

### 3.3. Vitamin D and Cardiovascular Risk

Several studies have demonstrated an essential link between vitamin D and CV risk (Table 2). Observational and prospective studies show that the prevalence of hypovitaminosis D is significantly higher in adults with T2DM when matched by sex and age to non-diabetics. These T2DM patients with hypovitaminosis D have significant thickening of the common carotid intima compared to those presenting normal serum 25(OH)D levels [49]. However, when 108 healthy postmenopausal women (mean age: 55 years) received a daily supplement containing minerals and vitamin D, no statistical significance was seen for intima–media thickness, compliance coefficient, or distensibility coefficient when compared to a placebo [50]. Oh and colleagues, in an in vitro study, found that 1,25(OH)2D suppressed macrophage foam cell formation in a cultured positive medium by reducing acetylated or oxidized LDL uptake. The study was designed using macrophages from 62 diabetic, hypertensive, and vitamin-D-deficient (<80 nmol/L) women (mean 55 years), and almost 50% less cholesteryl ester formation was seen when vitamin D was present in the medium [17].

The mechanisms by which the reduction in vitamin D concentrations could trigger T2DM are not fully understood. There is evidence that vitamin D directly stimulates insulin secretion by pancreatic β cells, and some other indirect actions have been proposed [62]. Additionally, considering the relevance of 25(OH)D in the regulation of the immune system, as suggested by the higher prevalence of hypovitaminosis D in patients with autoimmune diseases (e.g., Crohn’s disease, T2DM, rheumatoid arthritis, lupus, and multiple sclerosis) and some types of cancer (e.g., colon, prostate, breast, and pancreas), the possibility was also raised that exacerbation of inflammatory responses, which accompanies hypovitaminosis D states, may contribute to the worsening of insulin resistance [5,49,50,62,63].

Although observational studies indicate a positive association between vitamin D deficiency, reduction in HDL cholesterol/apolipoprotein A levels, and elevation of total cholesterol/triglycerides [64], recent clinical research has not observed significant benefits in plasma lipid concentrations with vitamin D supplementation [65]. Therefore, the consequences of hypovitaminosis D on the lipoprotein profile have yet to be established. Myrup and colleagues accompanied 74 postmenopausal healthy Danish women (>70 years) for 18 months. The protocol of supplementation with 0.5 μg of 1,25(OH)2D/day for 1 year presented no statistical significance for any of the parameters analyzed (i.e., total cholesterol (TC); high-density lipoprotein cholesterol (HDL-c); blood pressure at 0, 3, 6, 9, 12, and 18 months) [51]. On the other hand, a study of 463 postmenopausal women (45–75 years; mean age 58 years) showed that women with low 25(OH)D levels (<30 ng/mL) had higher levels of TC (*p* = 0.031), triglycerides (OR 1.55, 95%CI = 1.13–2.35), and HDL-c (OR 1.60, 95%CI = 1.19–2.40) [6].

Vitamin D supplementation has been found to improve several markers of cardiovascular health, including enhanced lipid profiles [57,59,66]. Thus, 25(OH)D deficiency is related to a significant worsening in TC, LDL, and triglyceride levels, as observed in a study reporting alterations of +9.4%, +13.5%, and +26.4%, respectively [57]. Vitamin D sufficiency has been shown to reduce TC, LDL, and triglycerides, with mean differences of −0.17, −0.19, and −0.12, respectively [59].

The link between AH and 25(OH)D deficiency is supported by prospective studies and some meta-analyses of observational studies [1,16]. Data from the National Health and Nutrition Examination Surveys (NHANES) from 2001 to 2006 showed an inverse association between serum 25(OH)D and systolic blood pressure after adjusting for age, gender, ethnicity, and BMI. More than 5000 adolescents aged 12 to 19 years were included in this study. Systolic blood pressure was significantly higher in the low-serum-25(OH)D group (serum 25(OH)D3 < 48.1 nmol/L) than in the medium (serum 25(OH)D 48.1 to <66.2 nmol/L) and high groups (serum 25(OH)D > 66.2 nmol/L) (109.8 ± 0.5 vs. 108.2 ± 0.4 and 108.4 ± 0.4 mmHg, respectively; *p* = 0.01) [56]. Similarly, in a study of over 1400 adolescents aged 13 to 15 years, it was found that 25(OH)D deficiency (serum 25(OH)D3 < 50 nmol/L) was associated with elevated levels of diastolic blood pressure. In this study, diastolic blood pressure was higher in the 25(OH)D-deficient group compared with the 25(OH)D3-sufficient group (*p* = 0.04) [61]. Other studies observed similar results, where increasing serum 25(OH)D levels resulted in significant decreases in systolic and diastolic blood pressure [58,67]. It was observed that for every 10 mmol/L increase in serum 25(OH)D there was an associated decrease in diastolic blood pressure (*p* = 0.02), independent of the BMI [58].

Studies demonstrate an inverse correlation between serum 25(OH)D levels and blood pressure—most evident when analyzing systolic blood pressure (SBP) in women over 50 years of age [68]—higher blood pressure levels in individuals with a history of less exposure to ultraviolet rays [69,70], and lower risk of AH in middle-aged women who ingest higher amounts of calcium/vitamin D through the consumption of milk and dairy products [71]. In 2001, Pfeifer and colleagues developed a study aiming to compare the effects of calcium (1.200 mg/day) versus calcium plus vitamin D supplementation (1.200 mg/day and 800 IU/day, respectively) on the SBP levels of postmenopausal women (n = 148; mean age = 74 years). Supplementation of vitamin D with calcium decreased SBP by 9.3% (*p* = 0.02) and heart rate by 5.4% (*p* = 0.02) when compared to the supplementation with calcium alone. Furthermore, 60 subjects (81%) in the calcium plus vitamin D group showed a decrease of 5 mmHg or more in SBP, compared with 35 (47%) subjects in the calcium group (*p* = 0.04) [52].

A large meta-analysis of prospective studies included more than 280,000 participants [13], including 55,816 patients with AH. The analysis included men and women with 25(OH)D levels measured at baseline. The participants with the highest levels of 25(OH)D were found to have a 30% lower risk of AH. Another study evaluated the relationship between 25(OH)D, PTH levels, and AH in the elderly [72]. In that study, vitamin D supplementation caused a reduction in PTH levels, which are a potentially modifiable determinant of blood pressure. Similar observations were described in a study that investigated 25(OH)D levels in patients with metabolic syndrome—low circulating 25(OH)D was associated with high PTH levels [73]. In that study, with more than 1000 adult participants, it was suggested that hyperparathyroidism might contribute to the worsening of metabolic syndrome and AH.

Over seven years, a prospective study found no significant differences in the adjusted mean changes in systolic or diastolic blood pressure by quartile of 25(OH)D. Incident AH was slightly and statistically significantly lower in the third quartile of 25(OH)D serum levels (48–<65 nmol/L) compared with the lowest quartile in 4863 healthy postmenopausal women (mean age = 66 years) [54].

In a prospective case–control study with 1484 middle-aged women, the authors observed a relative AH risk of 1.47 (95% CI: 1.10–1.97) for women with vitamin D deficiency compared to those with adequate serum levels [10]. The pathophysiological mechanisms suggested for this association include the elevation of serum PTH levels—directly related to the reduction in 25(OH)D—and the deleterious effects of hypovitaminosis D on activation of the RAAS and endothelial function [74].

The effects of PTH on blood pressure and CVD, as suggested by clinical observations [75] and by the induction of AH with the infusion of PTH, are probably mediated by the action of aldosterone. Indeed, a positive correlation between PTH and aldosterone levels was described in 2009 by Brunaud and colleagues, where several clinical and experimental studies showed that PTH could directly or indirectly stimulate (via the RAAS) the synthesis of mineralocorticoids [54,74,75]. It is essential to highlight that, in a large prospective study published in 2014, after the adjustment for potential confounders, the elevation of PTH—rather than the reduction in 25(OH)D concentrations—was associated with the risk of AH [27].

In the last decade, experimental studies with animals have shown that calcitriol is a potent suppressor of gene transcription that determines renin synthesis. Human studies have demonstrated that high calcitriol levels are associated with the suppression of plasma renin activity [76]. In fact, mice lacking vitamin D receptors have excessive plasma renin activity associated with AH [12]. These findings are of practical importance, since vitamin D replacement and the development of vitamin D analogs with negligible effects on calcium metabolism could suppress the RAAS, benefiting some patients with AH. However, the drug intervention studies currently available are insufficient to corroborate this benefit.

It is possible that elevations in plasma aldosterone also play a role in the endothelial dysfunction that has already been described in hypovitaminosis D states [53,55,60,77,78,79,80]. This hypothesis was supported by a large population analysis that demonstrated a positive correlation between aldosterone/renin levels and changes in endothelial function. Despite this, these results could be limited in middle-aged individuals due to the methods of assessing flow-mediated dilation of the brachialis. In this study, the vitamin D deficiency group (25(OH)D level of <20 ng/mL) had significantly higher prevalence of severe coronary artery disease (CAD) (53% vs. 38% *p* = 0.03), diffuse CAD (56% vs. 34% *p* = 0.03), and impaired endothelial-dependent vasodilation (FMD) (values < 4.5%; 50.6% vs. 7%; *p* < 0.002), as well as significantly lower flow-mediated dilation of the brachial artery (4.57% vs. 10.68%: *p* < 0.001) [80].

Recent in vitro studies also demonstrate that the benefits of vitamin D on endothelial function may be related to increased expression of the enzyme nitric oxide synthetase [81,82] and endothelial growth factor [83,84], inhibition of adhesion molecules, macrophage migration, and muscle cell proliferation [84,85], reduction in oxidative stress, and production of pro-inflammatory cytokines [32,79].

The effect of vitamin D replacement on endothelial function has been poorly investigated. Although some studies have not observed significant impacts of vitamin D supplementation on endothelial function, adhesion molecule production, or hs-CRP [32,85,86], the normalization of the production of ROS and the overexpression of the angiotensin II type 1 receptor in rats have already been documented [86]. Some controlled clinical studies have shown that vitamin D replacement can improve the parameters of vascular function [60]. Tarcin and colleagues (2009) presented significantly lower FMD in 25(OH)D-deficient subjects (25(OH)D < 25 nmol/l) than in controls (7.0 ± 3.2% vs. 11.2 ± 5.2%; *p* = 0.001), improved levels after replacement therapy (10.4 ± 3.3%; *p* = 0.002), and significantly lower post-treatment values of thiobarbituric-acid-reactive substances (TBARS) (4.7 ± 1.7 to 2.9 ± 0.7 ng/mg MDA; *p* = 0.0001) [60]. Therefore, even when analyzing this early sign of atherosclerosis and vascular injury, current evidence does not allow for estimating the real benefits of vitamin D replacement in the prevention or treatment of CVD, and further studies are needed.

## 4. Discussion

Oxidative stress is a transient or permanent disturbance in the state of oxidative equilibrium that generates regulatory changes within the cell, depending on the specific target and concentrations of ROS. Under regulatory conditions, ROS concentrations fluctuate in a controlled manner and are modulated by enzymatic and non-enzymatic antioxidant systems. Thus, there is a balance between oxidant and antioxidant activity. ROS levels increase if this homeostatic state fails, generating inflammation and dysfunctional physiological effects [46,48].

Oxidative imbalance has been associated with the progression of atherosclerosis and CV outcomes. Its increase impacts the development of several atherosclerotic risk factors, including vitamin D deficiency. Specific oxidative pathways involving pro-oxidant and antioxidant enzymes, such as nicotinamide adenine dinucleotide phosphate oxidase (NADPH), myeloperoxidase, SOD, and glutathione peroxidase, seem to play an essential role in the production of ROS. Vitamin D, in particular, is known to have the ability to reduce oxidative stress through the upregulation of cellular glutathione and SOD [44,46].

Observational and prospective studies demonstrate a correlation between hypovitaminosis D, increased levels of oxidative stress markers, and significant thickening of the common carotid intima—a common indicator of CV dysfunction. On the other hand, there is a lower risk of AH in individuals with normal serum levels of 25(OH)D who receive greater amounts of calcium/vitamin D through the consumption of milk and derivatives, for example. The pathophysiological mechanisms suggested for this association with the relative risk of developing AH include hypovitaminosis D’s deleterious effects on the RAAS’s activation and endothelial function [42,49,83].

The correlation between vitamin D deficiency and increased levels of oxidative stress markers is known. Therefore, vitamin D should be considered as an adjuvant therapy in the treatment of inflammatory diseases and/or cardiometabolic conditions. However, the effect of vitamin D replacement has still been scarcely investigated, and current evidence does not allow for estimating the actual benefits, dosages, and duration of treatment—especially for the prevention and/or treatment of CVD. More studies are also needed to investigate new strategies to modulate oxidative stress and to determine which lifestyle modifications should be implemented. When it comes to a nutritional approach, the Mediterranean diet and the Dietary Approach to Stop Hypertension are especially recommended by the American Heart Association as preventive or treatment approaches for CVD. A common feature of these dietary patterns is that they are rich in antioxidants. Therefore, adherence to healthy dietary patterns should always be investigated in patients with cardiometabolic disease [87,88,89].

## 5. Conclusions

Vitamin D supplementation could favor some risk groups, especially patients with chronic diseases, by reducing oxidative stress and endothelial damage. In fact, an increase in CVDs is observed with aging, and other conditions such as obesity, T2DM, AH, COVID-19, dyslipidemia, and smoking impair NO production, increasing RAAS activation and oxidative stress, which may contribute to endothelial dysfunction in these patients. Further studies analyzing the effects of vitamin D on endothelial function are of great interest, since the correction of hypovitaminosis D could minimize the damage to the endothelial function and, consequently, reduce the CV risk.

## Figures and Tables

**Table 1:** Vitamin D, oxidative stress and inflammation studies.

Author(s), Year, Reference Number	Characteristic of Study/Participants	Vitamin D Protocol	Oxidative Stress Parameters Analyzed	Results
Codoñer-Franch et al. (2012) [42]	Cross-sectional study (66 obese Caucasian children aged 7–14 year)	-	3-nitrotyrosine; sVCAM-1; IL-6	Inverse relationship between serum 25(OH)D levels with 3-nitrotyrosine (r = −0.424, *p* = 0.001) and sVCAM-1 (r = −0.272, *p* = 0.032). Comparing deficiency and sufficiency groups, IL-6 decreases significantly (18.0 [12.2–25.7] vs. 13.7 [10.7–19.3] pg/mL, *p* < 0.036).
Zhang et al. (2014) [43]	Cross-sectional study (1488 chinese children aged 7–11 year)	-	CRP; IL-6; MDA; SOD	Inverse association of serum 25(OH)D levels with CRP (1.21 ± 0.13 vs.1.04 ± 0.14), IL-6 (3.83 ± 0.33 vs. 3.58 ± 0.34), MDA (2.87 ± 0.21 vs. 2.83 ± 0.27), and SOD (95.38 ± 12.22 vs. 127.62 ± 15.98 U/mL, *p* < 0.001) comparing deficiency and sufficiency groups.
Akbari et al. (2018) [44]	Systematic review and meta-analysis of randomized (7 studies involving polycystic ovary syndrome women)controlled trials	Different vitamin D supplementation protocols (400–7142 IU/d)	CRP; MDA; NO; GSH	Vitamin D supplementation led to an increase in TAC, leading to a reduction in CRP (−1.03; 95%CI, −1.58, −0.49; *p* < 0.001) and MDA (−1.64, 95%CI −2.26 to −1.02, *p* < 0.001), however, there was no change in NO or GSH.
Mansournia et al. (2018) [45]	Meta-analysis (33 studies involving adults)	Different vitamin D supplementation protocols (10–11,200 IU/d)	CRP; MDA; NO; GSH; TAC	Supplementation significantly reduced CRP (WMD 0.27; 95%CI, −0.35, −0.20; *p* < 0.001) and MDA (WMD −0.43, 95%CI −0.62, −0.25, *p* < 0.001); and increased NO (WMD 4.33, 95%CI 0.96, 7.70), GSH (WMD 82.59, 95%CI 44.37, 120.81, *p* < 0.001) and TAC (WMD 57.34, 95%CI 33.48, 81.20, *p* < 0.001)
Sepidarkish et al. (2018) [46]	Meta-analysis (17 studies involving adults)	Different vitamin D supplementation protocols (300–20,000 IU/m)	TAC; GSH; MDA; NO	Supplementation in 13 clinical trials showed increased levels of serum TAC (SMD: 0.54 mmol/L, 95%CI: 0.29 to 0.79; I2 = 65.4%, *p* = 0.001) and GSH (SMD: 0.33; 95%CI: 0.11 to 0.54, *p* = 0.003; I2 = 61.2%, *p* = 0.001). MDA decreased significantly (SMD: −0.40 mmol/L, 95%CI: −0.60 to −0.21, *p* < 0.001) and NO was not significant different (0.17, 95%CI: −0.10 to 0.45, I2 = 71.1%, *p* = 0.21).
Cojic et al. (2021) [47]	Randomized Controlled Study (114 T2DM analyzed patients)	14,000 IU/wk vitamin D supplementation for 6 m. Vitamin D deficient group [25(OH)D ≤ 50 nmol/L]: initial correction of vitamin D levels 50,000 IU/wk for 3 m.	HbA1c; AOPP; HOMA-IR; MDA; CRP; TG/TBARS at 0, 3 and 6 m	Improved HbA1c levels over the 3-month and 6-month period. Significant decrease inAOPP levels over the 3-month period. HOMA-IR, MDA and TG/TBARS was not statistically significant. The inflammatory marker—CRP had a decrease but not statistically significant.
Moslemi et al. (2022) [48]	Meta-analysis (23 studies involving adults)	Different vitamin D supplementation protocols (100–30,708 IU/d)	CRP; TNF-α; MDA; IL-6; TAC; GSH	Supplementation significantly reduced CRP (ES = −0.42; 95%CI: −0.55, −0.29, *p* < 0.001), TNF-α (ES = −0.27; 95%CI: −0.42, −0.12; *p* < 0.001), MDA concentrations (ES = −0.37; 95%CI: −0.48, −0.25, *p* < 0.001). No significant difference at IL-6 levels (ES = −0.35, 95%CI: −0.80, 0.10; *p* = 0.125), TAC (ES = 0.68; 95%CI: −0.31, 1.66, *p* = 0.179), GSH activity (ES = 0.08; 95%CI: −0.44, 0.60, *p* = 0.757)

Abreviations: 25-hydroxyvitamin D (25(OH)D); soluble Vascular Cell Adhesion Molecule-1 (sVCAM-1); Interleukin-6 (IL-6); Serum C-reactive protein (CRP); Malondialdehyde (MDA); Superoxide dismutase (SOD); Nitric oxide (NO); Glutathione (GSH); Total antioxidant capacity (TAC); Glycated haemoglobin (HbA1c); Advanced oxidation protein products (AOPP); Insulin resitance index (HOMA-IR); Tryglicerides/Thiobarbituric acid-reactive substances (TG/TBARS); Tumor necrosis factor-α (TNF-α); Type 2 Diabetes Mellitus (T2DM); Month (m); Week/s (wk); Year/s (yr); Day/s (d). Red color: Inflammatory markers.

**Table 2:** Vitamin D and CVD risk studies.

Author(s), Year, Reference Number	Characteristic of Study/Participants	Vitamin D Protocol	CVD Parameters Analyzed	Results
Myrup; Jensen; McNair (1992) [51]	Double-blind, randomized study/74 healthy Danish women (>70 year)	1 year of dailly 0.5 μg 1,25(OH)2D supplementation	TC; HDL; BP at 0,3,6,9,12,18 m.	No statistical significance for all parameters.
Pfeifer et al. (2001) [52]	Double-blind, randomized, placebo-controlled study/148 (25(OH)D serum < 50 nmol/L) women (>70 year; mean 74 year)	8 wk of dailly supplementation of calcium 1.200 mg plus 800 IU of vitamin 1.25(OH)2D or just calcium 1.200 mg.	SBP; DBP; HR at 0 and 8 wk.	Compared with calcium, supplementation with vitamin D3 plus calcium decrease SBP in 9.3% (*p* = 0.02) and decrease HR in 5.4% (*p* = 0.02). Sixty subjects (81%) in the vitamin D3 plus calcium group compared with 35 (47%) subjects in the calcium group showed a decrease in SBP of 5 mmHg or more (*p* = 0.04)
Braam et al. (2004) [50]	Randomized placebo-controlled study/108 healthy women (>50 year and <60 year; mean 55 year)	3 year placebo or a daily supplement containingminerals and vitamin D.	CC; DC; IMT; E at 0 and 3 yr.	No statistical significance for all parameters.
Targher et al. (2004) [49]	Case-control study/ 390 diabetes patients (130 with vitamin D hypovitaminosis: 25(OH)D ≤ 37.5 nmol/L) and 390 matched-control individuals. (mean 58 year)	Compared winter serum [25(OH)D] concentrations in 390consecutive type 2 diabetic patients and 390 nondiabetic controlswho were comparable for age and sex.	Prevalence of hypovitaminosis; IMT; HbA1c; Fibrinogen; CRP	In diabetic patients, prevalence of hypovitaminosis D was higher (34.0 vs. 16.4%, *p* < 0.001)and significantly higher IMT (1.10 ± 0.15 vs. 0.87 ± 0.14 *p* < 0.001), HbA1c (7.5 ± 1.3 vs. 7.2 ± 1.4 *p* < 0.05), fibrinogen (4.69 ± 0.8 vs. 4.21 ± 0.9 *p* < 0.001)and CRP (4.98 ± 6·5 vs. 4.30 ± 6.1 *p* < 0.001) concentrations was seen in these condition.
Bolland et al. (2009) [53]	Randomized placebo-controlled study/1471 (25(OH)D serum >25 nmol/L) healthy women (>55 year; mean 74 year) supplementing calcium	-	Incidence of stroke; MI; CA; HF; adverse BP; TC at 0 and 5 yr.	No statistical significance for all parameters comparing deficient (<50 nmol/L serum 25OHD) and non-deficient (>50 nmol/L serum 25OHD) at 0 and 5 yr.
Oh et al. (2009) [17]	In vitro study/Macrophage of 62 diabetics, hypertensive and vitamin D deficient (<80 nmol/L) patients (mean 55 year)	Same patient’s macrophages were cultured in vitamin D deficient or in 1,25(OH)2D3 medium and exposed to modified LDL.	Foam cell formation.	1,25(OH)2D suppressed foam cell formation by reducing acetylated or oxidized LDL uptake - almost 50%less cholesteryl ester formation.
Margolis et al. (2012) [54]	Longitudional study/4863 healthy women (50–79 yr; mean 66 year)	-	SBP; DBP	Over 7 yr, there were no significant differences in the adjusted mean change in systolic or diastolic blood pressure by quartile of 25(OH)D. Incident hypertension was slightly and statistic significantly lower in the third quartil of 25(OH)D (48– < 65 nmol/L) compared with the lowest quartile.
Syal et al. (2012) [55]	Observational study/100 Indian individuals - 81% male (37–75 yr, mean 57 year)	-	Prevalence of severe CAD; diffuse CAD; FMD; brachial artery FMD	Vitamin D deficiency group (25(OH)D level of <20 ng/mL) had significantly higher prevalence of severe CAD (53% vs. 38% *p* = 0.03), diffuse CAD (56% vs. 34% *p* = 0.03), impaired FMD (values < 4.5%; 50.6% vs. 7%; *p* < 0.002) and significantly lower brachial artery flow-mediated dilation (4.57% vs. 10.68%: *p* < 0.001).
Schmitt et al. (2018) [6]	Observational, cross-sectional cohort study/463 women (45–75 yr; mean 58 year)	-	TC; Triglycerides; HDL.	Women with low 25(OH)D levels (<30 ng/mL) had higher TC (*p* = 0.031); triglycerides (OR 1.55, 95%CI = 1.13–2.35) and low HDL levels (OR 1.60, 95%CI = 1.19–2.40).
**Children, Adolescents and Young Adults Studies**				
Ganji et al. (2011) [56]	Observational, cross-sectional cohort study/5867 children and adolescents (12–19 year). Serum 25(OH)D: low (<48.1 nmol/mL), medium (48.1 to <66.2 nmol/L), and high (>66.2 nmol/L).	-	SBP	SBP significantly higher in the low serum 25(OH)D group than in the medium group and high groups (109.8 ± 0.5 vs. 108.2 ± 0.4 and 108.4 ± 0.4 mmHg, *p* = 0.01)
Lupton et al. (2016) [57]	Observational, cross-sectional cohort study/20,360 adults. Serum 25(OH)D: deficient (<20 ng/mL), intermediate (≥20–30 ng/mL), and optimal (≥30 ng/mL).	-	TC; Triglycerides; LDL.	25(OH)D deficiency is related to a significant worsening in TC, LDL, and triglycerides levels observed in a study reporting alteration of +9.4%, +13.5%, and +26.4%, respectively
Petersen et al. (2015) [58]	Observational, cross-sectional cohort study/782 children (8–11 year).	-	DBP	For every 10 mmol/L increase in serum 25(OH)D there is an association with a decrease in DBP (*p* = 0.02), independent of the BMI.
Skaaby et al. (2012) [59]	Prospective Study/ 4330 adults. Serum 25(OH)D: sufficiency (> 48.0nmol/l).	-	TC; Triglycerides; LDL.	Vitamin D sufficiency has been shown to reduce TC, LDL, and triglycerides with mean differences of −0.17, −0.19, and −0.12, respectively
Tarcin et al. (2009) [60]	Case-control study/23 asymptomatic vitamin D-deficient (25(OH)D < 25 nmol/l) and 23 matched-control individuals (mean 25(OH)D level of 75 nmol/l). (mean 23 year)	Vitamin D-deficient group: 300,000 IU/mintramuscular for 3 months	FMD; TBARS	Significantly lower FMD in 25(OH)D-deficient subjects than controls (7.0 ± 3.2% vs. 11.2 ± 5.2%; *p* = 0.001), improved after replacement therapy (10.4 ± 3.3%; *p* = 0.002). Significantly lower post treatment values of TBARS (4.7 ± 1.7 to 2.9 ± 0.7 ng/mgMDA; *p* = 0.0001).
Tomaino et al. (2015) [61]	Observational, cross-sectional cohort study/1441 adolescents (13–15 year). Serum 25(OH)D: deficiency (serum 25(OH)D3 < 50 nmol/L).	-	DBP	DBP higher in the 25(OH)D-deficient group compared with the 25(OH)D3-sufficient group (*p* = 0.04)

Abreviations: 1,25-dihydroxyvitamin D(1,25(OH)2D); Total cholesterol (TC); High density lipoprotein (HDL); Blood pressure (BP); 25-hydroxyvitamin D (25(OH)D); Sistolic blood pressure (SBP); Diastolic blood pressure (DBP); Heart rate (HR); Flow-mediated dilation (FMD); Thiobarbituric acid-reactive substances (TBARS); Compliance coefficient (CC); Distensibility coefficient (DC); Intima-media thickness (IMT); Glycated haemoglobin (HbA1c); Serum C-reactive protein (CRP); Young’s Modulus (E); Myocardial infarction (MI); Cancer (CA); Heart failure (HF); Coronary artery disease (CAD); Low density lipoprotein (LDL); Month (m); Week/s (wk); Year/s (yr); Day/s (d). For convertion of 25(OH)D from ng/ml to nmol/L, multiply by 2.496.

## Data Availability

Not applicable.

## List of Abbreviations

[25(OH)D]	25-Hydroxyvitamin D
[1,25(OH)2D]	1,25-Dihydroxyvitamin D
AH	Arterial hypertension
BMI	Body mass index
CAD	Coronary artery disease
COVID-19	Coronavirus disease 2019
CRP	*C*-reactive protein
CVD	Cardiovascular disease
CV	Cardiovascular
DM	Diabetes mellitus
FMD	Endothelial-dependent vasodilation
GSH	Glutathione
HA	Hypertension
HDL-c	High-density lipoprotein cholesterol
hs-CRP	High-sensitivity *C*-reactive protein
IL-4	Interleukin-4
IL-6	Interleukin-6
IL-10	Interleukin-10
IR	Insulin resistance
MDA	Malondialdehyde
MS	Metabolic syndrome
NADPH	Nicotinamide adenine dinucleotide phosphate oxidase
NHANES	National Health and Nutrition Examination Surveys
NO	Nitric oxide
PTH	Parathyroid hormone
PCOS	Polycystic ovary syndrome
RAS	Renin–angiotensin system
RAAS	Renin–angiotensin–aldosterone system
ROS	Reactive oxygen species
SBP	Systolic blood pressure
SOD	Superoxide dismutase
sVCAM-1	Soluble vascular cell adhesion molecule-1
TAC	Antioxidant capacity levels
TBARS	Thiobarbituric-acid-reactive substances
TC	Total cholesterol
T2DM	Type 2 diabetes mellitus
TNF	Tumor necrosis factor
